# Precise Position Estimation Using Smartphone Raw GNSS Data Based on Two-Step Optimization

**DOI:** 10.3390/s23031205

**Published:** 2023-01-20

**Authors:** Taro Suzuki

**Affiliations:** Future Robotics Technology Center, Chiba Institute of Technology, Chiba 2750016, Japan; taro@furo.org

**Keywords:** GPS, GNSS, smartphone, localization, time-differenced carrier phase

## Abstract

This paper presents a high-precision positioning method using raw global navigation satellite system (GNSS) observations from smartphones in the Google smartphone decimeter challenge (GSDC). Compared to commercial GNSS receivers, smartphone GNSS observations are noisy owing to antenna limitations, making it difficult to apply conventional high-precision positioning methods. In addition, it is important to exclude outliers in GSDC because GSDC includes data in environments where GNSS is shielded, such as tunnels and elevated structures. Therefore, this study proposes a smartphone positioning method based on a two-step optimization method, using factor graph optimization (FGO). Here, the velocity and position optimization process are separated and the velocity is first estimated from Doppler observations. Then, the outliers of the velocity estimated by FGO are excluded, while the missing velocity is interpolated. In the next position-optimization step, the velocity estimated in the previous step is adopted as a loose state-to-state constraint and the position is estimated using the time-differenced carrier phase (TDCP), which is more accurate than Doppler, but less available. The final horizontal positioning accuracy was 1.229 m, which was the first place, thus demonstrating the effectiveness of the proposed method.

## 1. Introduction

High-precision positioning with smartphones has become important for various applications, such as pedestrian navigation, vehicle lane-level navigation, and the increasing number of location-based games and virtual reality technologies. GNSS is conventionally used for location estimation in outdoor environments and as a means of location estimation in smartphones as it can estimate absolute positions on the Earth provided signals can be received from a satellite. Although smartphones have long been equipped with GNSS, users have only been able to access location information output using the GNSS receivers of smartphones. In 2016, the Android operating system released an application program interface to access raw GNSS measurement data from GNSS installed in smartphones [1]. Accordingly, raw GNSS data (pseudorange, pseudorange rate (Doppler), and accumulated delta range (carrier phase)), could be acquired. This facilitated the development of position-estimation algorithms for smartphones using raw GNSS measurements. Consequently, high-precision positioning at the decimeter and centimeter levels on smartphones has garnered significant attention [2,3,4,5].

However, there are several challenges associated with smartphone positioning compared with positioning using commercial GNSS receivers [4,5]. The GNSS antennas of smartphones exhibit lower performance than those of GNSS receivers for surveying and the noise in GNSS observations is very significant. Consequently, the application of existing high-precision positioning methods to smartphones, such as precise point positioning (PPP) [6,7] and real-time kinematic (RTK) GNSS [8,9], is challenging. The usual positioning accuracy of a smartphone is approximately 3–10 m, which results in significant challenges for advanced navigation and other applications, such as sidewalk- and lane-level navigation for pedestrians and vehicles.

To address this challenge, in 2020, Google released a dataset with GNSS raw measurements acquired by Android smartphones to develop positioning algorithms using smartphones [10]. Furthermore, in 2021, Google launched the Google smartphone decimeter challenge (GSDC) to further accelerate the development of high-precision positioning technology for smartphones [11]. In 2022, GSDC 2022 was held from 3 May to 30 July 2022, using a new dataset based on the raw GNSS measurements of smartphones [12]. This paper describes the winning solution at GSDC 2022.

## 2. Related Researches

Various high-precision positioning methods using GNSS measurements from smartphones have been studied. When applying PPP or RTK-GNSS to smartphones, noise from the built-in GNSS antenna of the smartphone becomes a problem [13]. The methods of installing a smartphone on a choke ring antenna [14,15], or using a re-radiating antenna or an external antenna [16,17], can achieve high-accuracy positioning with centimeter accuracy. However, PPP and RTK-GNSS are not effective when a smartphone is mounted on the dashboard of a vehicle and GNSS data is acquired by an internal antenna, as in the case of GSDC. Some research has been conducted to improve the accuracy of RTK-GNSS and PPP by combining GNSS observations from smartphones and IMUs [18,19]. However, time synchronization between Android-based IMU and smartphone’s GNSS is an issue.

Graph-based optimization has been extensively studied in robotics; however, it has recently been actively studied in the GNSS field. The factor graph optimization (FGO) was proposed to model factorizations [20]. FGO can apply various complex non-linear constraints and simultaneously optimize all state variables (the entire driving trajectory). A factor graph is a graphical representation in which there is an unknown state variable (variable nodes) and a factor (factor nodes) that is a function of the state variable. The edges connecting the factor and variable nodes can be considered as constraints on the state variable by the factor. Hence, the state estimation problem is reduced to an optimization problem using the objective function constructed by the factor graph representation. One of the earliest studies proposed a robust optimization method using only GPS pseudorange observations based on factor graph optimization [21], which improved positioning accuracy better than least-squares-based positioning. Subsequently, extensions to real-time position estimation [22] and combinations with IMU and other sensors have been studied [23,24,25]. In [26], the authors discussed a comparative evaluation of GNSS positioning based on graph-based optimization with that based on least-squares-based positioning and extended Kalman filter. Several applications of time-differenced carrier phases (TDCP) to FGO have also been studied [27,28,29]. The study by [30] implemented precise point positioning using carrier phase measurements with graph optimization and compared it with Kalman-filter-based implementation. The results indicated that factor graph-based optimization exhibited better performance than conventional filtering methods, such as the extended Kalman filter. This is due to the global optimization with all the observations from past to present in the graph structure.

In this study, we propose a method to estimate location using FGO based on GNSS observations of smartphones, especially TDCP. The performance of positioning by graph-based optimization significantly depends on the graph structure. In most of the previous studies, the pseudorange of GNSS is adopted for graph construction. In addition, the FGO target is commercial GNSS receivers and GNSS observations of smartphones are yet to be used. This study is unique in that it adopts TDCP for graph optimization using GNSS observations from smartphones. Its optimization process is divided into two steps.

## 3. GSDC 2022 Overview

### 3.1. Dataset

At the GSDC, each dataset included raw GNSS measurements collected by several Android smartphone devices, together with the ground truth trajectories collected by a high-grade GNSS and an inertial navigation unit (IMU) integration system for reference [10,11]. The log data of multiple smartphones mounted on the dashboard of a vehicle were provided in two datasets: training data with reference position and test data for evaluation in the competition. The smartphone logs included observations such as the GNSS pseudorange, pseudorange rate, accumulated delta range, etc. Furthermore, the smartphone’s IMU data, such as acceleration and angular velocity, were also included in the log data.

**Figure 1:**
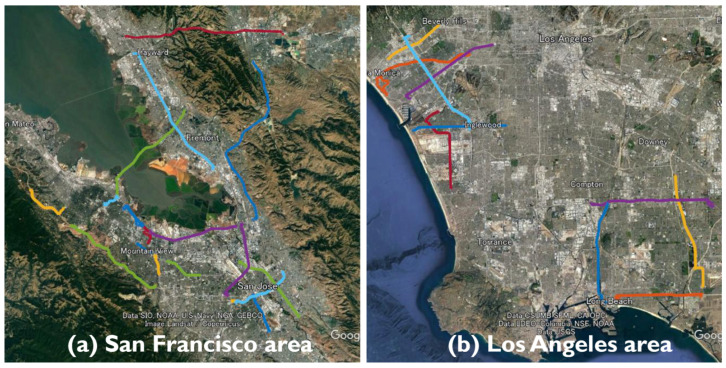
Driving trajectories included in test data provided at GSDC 2022. In total, 36 runs were provided and divided into two parts: one run in the San Francisco area and another in the Los Angeles area.

Driving data for five types of smartphones (Google Pixel4, Google Pixel5, Google Pixel6 Pro, Samsung Galaxy S20 Ultra, and Xiaomi Mi8) were provided at the GSDC 2022. Although the driving data for the San Francisco area was provided at GSDC 2021, that for the Los Angeles area was included at GSDC 2022. Figure 1 presents the GSDC 2022 data from the 36 runs included in the test dataset. At GSDC 2021, each run included data acquired simultaneously by multiple smartphone models; however, at GSDC 2022, only one type of smartphone was used for each run. This implies that the technique of improving accuracy by assembling the positions estimated independently for each smartphone was no longer available. All smartphones had a built-in dual-frequency GNSS receiver chip and included dual-frequency L1 and L5 observations relative to GPS and Galileo.

**Figure 2:**
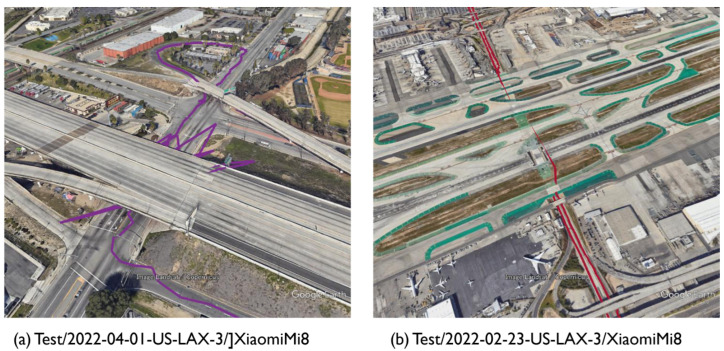
Los Angeles vehicle driving trajectory included in GSDC 2022. (**a**) Stops under elevated tracks and (**b**) runs through long tunnels where GNSS signals are completely blocked.

The driving data provided can be divided into two categories: highway driving in an open-sky environment and street driving in an area lined with trees and buildings. The Los Angeles driving data included several long tunnels and elevated sections, including several 10 s GNSS signal blockages. In approximately half of the runs included in the test data, the data were run on different courses not included in the training data. Figure 2 illustrates a section of the Los Angeles travel trajectory in the test data that stops under an elevated track or travels in a tunnel. There are certain sections where receiving GNSS signal is difficult, which results in large outliers and missing data.

### 3.2. Score Metric

In the GSDC, the trajectories of all smartphones in the test dataset are estimated and evaluated using a score according to their accuracies. Here, the score is computed via the mean of the 50th and 95th percentile of the horizontal positioning errors. The estimated latitudes $\Phi_{i}$ and longitudes $\Lambda_{i}$ of the *i*-th smartphone trajectory can be defined as follows.

(1) $$\Phi_{i}=\left\{\phi_{1}\cdots \phi_{M}\right\},\;\Lambda_{i}=\left\{\lambda_{1}\cdots \lambda_{M}\right\}$$

where *M* denotes the total epochs of the smartphone’s trajectory. $D_{i}=\left\{d_{1}\cdots d_{M}\right\}$ represent the horizontal errors of the trajectory computed from the Haversine formula [31]. The horizontal error of *j*-th epoch $d_{j}$ is represented as follows.

(2) $$d_{j}=harversine\left(\left(\phi_{j},\lambda_{j}\right),\left(\phi_{GT,j},\lambda_{GT,j}\right)\right)$$

where harversine is a function that calculates the Haversine distance between two sets of latitudes and longitudes, while the subscript GT represents the ground truth. The score for *N* trajectories is calculated as follows.

(3) $$score=\frac{1}{N}\sum_{i=1}^{N}\frac{percentile\left(D_{i},50\right)+percentile\left(D_{i},95\right)}{2}$$

where percentile(D,p) computes a value that is greater than *p* percent of the values in *D*. Note that the altitude of the smartphone is ignored. In addition, the smartphone positioning can exhibit significantly large errors owing to multipath; however, in the afore-presented metric, the large top 5% positioning errors are ignored and do not affect the score.

### 3.3. Baseline Position

In addition to the raw GNSS data, the competition hosts provided a “baseline” of location estimation results for each smartphone. This baseline is the result of least-squares-based processing of the pseudorange observed by the smartphone in estimating the position. Table 1 presents the scores for the two categories of driving data in the GSDC 2022 training dataset. The highway represents an open-sky environment. The 3.6 m score is practical for positioning with pseudorange. However, in street areas, GNSS signals were blocked by roadside trees, which increased the positioning error to 4.6 m.

The satellite observation performance of smartphones has the following characteristics. First, smartphones cannot track satellite signals stably and only a small number of satellites can be observed continuously with dual-frequency signals. In addition, regarding satellite signal quality, satellites observed by smartphones generally exhibit a low carrier-to-noise ratio [5,32]. Furthermore, the positioning accuracy of smartphones is significantly impaired by multipath. The observed pseudorange of a smartphone is substantially noisier than that of a commercial GNSS receiver and GNSS code positioning using only the pseudorange has limited positioning accuracy.

## 4. Strategy

From the exploratory data analysis, GNSS observations, such as pseudorange and Doppler shift, from smartphones are more significantly noisy than commercial GNSS receivers. In addition, owing to the limitations of antennas on smartphones, the real-time kinematic (RTK)-GNSS technique, which is usually used for high-precision positioning, is difficult to utilize because it is difficult to solve the integer ambiguities in the carrier phase measurements using smartphone antenna. In addition, missing data, abnormal values, and unsynchronized observation time for each smartphone exist. Even in an open-sky environment, a 3-m accuracy cannot reach the decimeter level. Hence, we have to improve the absolute position accuracy beyond the limits of pseudorange accuracy. Because excessive noise and several outliers exist in the observations, a robust position-estimation method is required. Finally, because GSDC targets high-precision positioning by post-processing, methods specific to post-processing are beneficial.

At GSDC 2021, the author proposed a location estimation method based on smartphone observations using global optimization with FGO [33]. Here, the method developed in 2021 was modified to improve the location estimation accuracy, particularly in environments where the accuracy of pseudorange deteriorates under trees and elevated structures. The pseudorange of smartphones is noisy; hence, achieving highly accurate position estimation using the pseudorange alone is challenging. However, if the carrier phase can be tracked continuously, the relative position change (velocity) can be estimated with high accuracy from the TDCP [34]. Because of the limited availability of valid TDCPs, velocity can be estimated from more robust Doppler observations, although they are less accurate than TDCP. However, Doppler observations remain unavailable when satellites are shielded for long periods, such as in elevated structures or tunnels.

**Figure 3:**
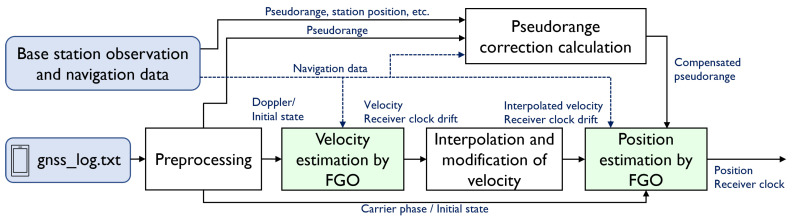
Flow of the proposed method comprising two optimization steps: velocity estimation and position estimation via factor graph optimization (FGO).

Therefore, we adopted a two-step optimization to estimate the position of the smartphone. Figure 3 illustrates the flow of the proposed method. First, we estimated the velocity, where the 3D velocity of the smartphone and receiver clock drift were estimated by employing FGO with Doppler observations, which are readily available but less accurate. From the 3D velocity estimated by the optimization, outliers in velocity were detected, excluded, and interpolated to obtain an estimate of the 3D velocity continuously. Subsequently, the states to be optimized were the 3D position and receiver clock bias of each satellite system. The 3D velocity and clock drift obtained in the previous step were adopted as loose constraints between the states in this step. In addition, if valid TDCPs were obtained, TDCP-based constraints between the states were added and, as an absolute position constraint, the pseudorange of the smartphone, whose error was corrected using the GNSS base-station, was utilized. Even when no TDCP was available, this method added a state-to-state constraint based on the velocity estimated using Doppler in the first step and the position could be obtained even in the tunnel.

## 5. Preprocessing

In the preprocessing step, the initial values of the optimized state to be determined, including the GNSS pseudorange, carrier phase, and Doppler observations, are calculated from the smartphone logs. Here, various filtering processes are adopted to discard invalid GNSS observations and reject unreliable observations. Because smartphone observations contain larger amounts of unreliable data than commercial receivers, it is very important to screen GNSS observations in preprocessing.

### 5.1. Initial State Estimation

The estimated states in the optimization step are the 3D position and velocity in the Earth-centered Earth-fixed (ECEF) coordinate system and the receiver clock bias and drift. These initial values need to be pre-determined before optimization.

Regarding the initial 3D position for the optimization, the baseline position is utilized. However, the baseline position contains some large jumps and missing parts; hence, the following process is applied.

1 Convert to the east-north-up (ENU) coordinate system and detect large jumps in the altitude.
2 Delete the epoch detected in Step 1 as an outlier.
3 Interpolate the 3D position of missing epochs from the previous and next data.

The above process determines the initial values of 3D positions for all epochs. The initial value of 3D velocity is determined by simply calculating the difference in the initial 3D positions.

For the initial clock bias and drift, the log data from the smartphone contains estimates of the built-in GNSS receiver clock bias and drift, which are adopted as the initial values. Note that, among the smartphones, for the XiaomiMi8, the difference between the estimated clock bias is adopted as the initial value of the clock drift, because the clock drift is an invalid value. The missing data are also complemented with the previous and next data to calculate the initial values of the clock and clock bias for all epochs.

### 5.2. Selection of GNSS Observations

Similar to normal GNSS positioning, the satellite elevation angle and carrier-noise ratio (CNR) masks are used to reject all observations (pseudorange, Doppler, and carrier phase) for satellites with a low elevation angle and low signal strength. Here, the values of the satellite elevation angle and CNR masks are tuned and determined for each run. If the multipath indicator (MultipathIndicator) in the smartphone log is turned on, the observations for that satellite are rejected. The following is a description of the process for each observation.

#### 5.2.1. Pseudorange Selection

Similar to [35], the observation values of valid GNSS signals are selected based on the pseudorange tracking status contained in the smartphone logs. In addition, the pseudorange residuals are calculated using the initial 3D position and clock bias described above. Thresholding in the pseudorange residual rejects pseudorange observations with large multipath errors.

#### 5.2.2. Doppler Selection

Doppler observations (or pseudorange rates) $\dot{\rho }$ are the most robust observations in smartphones, even in adverse environments. To circumvent large errors in Doppler observations, the residuals of Doppler observations are calculated using the initial 3D velocity and clock drift and the aforementioned pseudorange; in addition, the Doppler observation values with large errors are rejected by thresholding.

#### 5.2.3. Carrier Phase Selection

**Figure 4:**
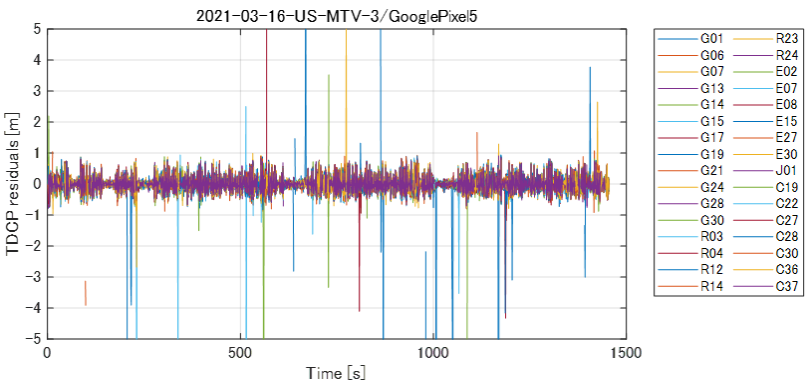
TDCP residual of L1 signals computed by Equation (4). The Doppler observation is used to detect and exclude cycle slips in the carrier-phase observation.

From the carrier-phase tracking status, the carrier phases continuously tracked with no cycle slip are reserved [35]. However, even in the carrier phases that have passed these processes, some carrier phases that contain cycle slips or errors still remain. Therefore, by comparing the Doppler observation and the aforementioned carrier phase, the outliers of the carrier phase are rejected by checking the consistency. The consistency of the carrier phase of the satellite *k* at the *i*-th $\phi_{i}^{k}$ and i+1-th epochs $\phi_{i+1}^{k}$ can be calculated from Doppler observation (in m/s) ${\dot{\rho }}_{i}^{k}$ and ${\dot{\rho }}_{i+1}^{k}$ as follows.

(4) $$\left|\lambda \left(\phi_{i+1}^{k}-\phi_{i}^{k}\right)-\frac{\left({\dot{\rho }}_{i}^{k}+{\dot{\rho }}_{i+1}^{k}\right)}{2}\Delta t\right|<D_{th}$$

where, λ, Δt, and $D_{th}$ denote the wavelength, time step (1 s in the GSDC), and threshold value, respectively. The TDCP residuals of L1 signals in Equation (4), illustrated using the actual smartphone observations in the training dataset, are presented in Figure 4. The colors of the plots in Figure 4 represent different satellites and the increase in the residuals due to cycle slips in the carrier phase can be observed. Here, we used 1 m as the threshold value of $D_{th}$ to exclude cycle slips.

#### 5.2.4. Example of Observation Selection

**Figure 5:**
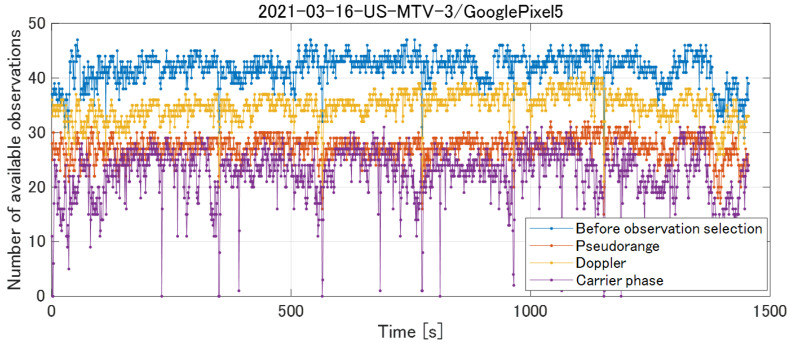
Selection of GNSS observations by preprocessing. Compared to the carrier phase, Doppler is highly available.

Using the aforementioned GNSS observation selection method, we preprocessed GNSS observations for the runs included in the GSDC 2022 training dataset. Figure 5 presents the change in the number of observations before and after selection of the GNSS pseudorange, Doppler, and carrier phase. The number of observations decreases significantly after the selection of the GNSS observations, thus verifying that the raw GNSS observations from smartphones contain a large amount of unreliable data with large errors. In addition, Doppler observations are more available than carrier-phase observations, and can be used even during times when the carrier phase contains cycle slips and is unavailable. The proposed method first estimates the 3D velocity using Doppler observations that are robust to adverse GNSS reception environments and then estimates accurate positions using less available, but more accurate, carrier phases in the next step.

## 6. Velocity-Estimation Step

In the velocity-estimation step, the state V estimated in epoch *i* was the 3D velocity and receiver clock drift. The state V can be expressed as:

(5) $$V_{i}=\left[\begin{array}{c} v_{i}\\ {\dot{t}}_{i} \end{array}\right]$$

(6) $$v_{i}={\left[\begin{array}{ccc} v_{x,i} & v_{y,i} & v_{z,i} \end{array}\right]}^{T}\;{\dot{t}}_{i}={\dot{t}}_{\mathrm{gpsL}1,i}$$

where ${\dot{t}}_{\mathrm{gpsL}1,i}$ represents the receiver clock drift relative to the GPS time in m/s. The 3D velocity $v_{i}$ is represented in the ECEF coordinate system. Here, only the clock drift computed from the GPS L1 signal was included in the state because the inter-system bias, including the receiver clock bias, between GPS L1 and other satellite systems can be considered constant.

**Figure 6:**
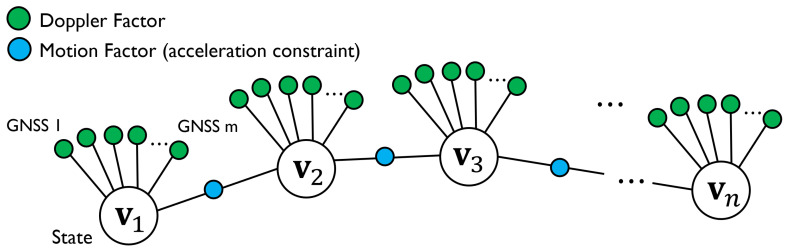
Graph structure of proposed method in velocity-estimation step. The Doppler and motion factors are adopted to estimate the velocity.

The graph structure of the proposed FGO is presented in Figure 6. The circled markers in the figure represent the factor nodes, where the variable nodes are connected to the Doppler factors from each satellite and the motion factor is connected between variable nodes as an acceleration constraint.

### 6.1. Doppler Factor

The observed Doppler frequency can be converted to a pseudorange rate. GNSS pseudorange rate from the Doppler of the satellite *k* in the *i*-th epoch $\rho_{i}^{k}$ can be modeled as follows.

(7) $${\dot{\rho }}_{i}^{k}=u_{i}^{k}\left(v_{s,i}^{k}-v_{i}\right)+{\dot{t}}_{i}$$

where $v_{s,i}^{k}$ represents the satellite velocity in the ECEF coordinate system. $u_{i}^{k}=\left[u_{x,i}^{k},u_{y,i}^{k},u_{z,i}^{k}\right]$ denotes the unit line-of-sight vector from the receiver to the satellite *k* in ECEF.

The error function of the Doppler factor is represented as follows.

(8) $$e_{d,i}^{k}=H_{v,i}^{k}V_{i}-\left({\dot{\rho }}_{i}^{k}-u_{i}^{k}v_{s,i}^{k}\right)$$

Here, the measurement matrix $H_{v,i}^{k}$ can be formulated as:

(9) $$H_{v,i}^{k}=\left[\begin{array}{cc} u_{i}^{k} & 1 \end{array}\right]$$

Together with the error function, the optimization utilizes the information matrix $\Omega_{d,i}^{k}$, which represents the accuracy of the Doppler observations. Here, the information matrix is determined from a simplified model, where the error depends on the satellite elevation angle.

### 6.2. Motion Factor

The variable nodes in the graph are the velocity and clock drift and the constraints between the nodes function as acceleration constraints. Although acceleration observations can be obtained from the smartphone’s IMU, they were not utilized in this study because they require a coordinate transformation based on the smartphone’s orientation, including synchronization with GPS time. The motion factor uses the assumption that the velocity does not change significantly (i.e., acceleration is small) between successive variable nodes. The error function was simply defined as acceleration being close to zero, such that the velocity changed smoothly, as follows:

(10) $$e_{m,i}=V_{i+1}-V_{i}$$

Here, by appropriately providing $\Omega_{m,i}$, the information matrix during optimization based on the maximum value of the vehicle’s actual acceleration, large jumps in the velocity estimated by optimization can be suppressed and a continuous velocity can be estimated.

### 6.3. Optimization

The objective function to be optimized is presented as follows.

(11) $$\hat{V}=\underset{V}{\mathrm{argmin}}\sum_{i}{∥e_{m,i}∥}_{\Omega_{m,i}}^{2}+\sum_{i}\sum_{k}{∥e_{d,i}^{k}∥}_{\Omega_{d,i}^{k}}^{2}$$

The M-estimator was used to reject the multipath error of Doppler measurements during the optimization [27]. The Huber function was adopted as the influence function of the M-estimator. The Huber function works remarkably for GNSS observations with several outliers, as adopted in [28,36]. The hyperparameters of the Huber function are determined by trial and error and the same values are used for all runs. Furthermore, the GTSAM [37] was utilized for the graph optimization backend, while RTKLIB [38] was used for GNSS general computation.

### 6.4. Outlier Removal and Interpolation

Interpolation was performed to discard outliers in the estimated velocity via FGO and also to obtain velocity estimates at all epochs. The estimated velocities in the ECEF coordinate system were transformed to the ENU coordinate system. In addition, the threshold for the estimated velocity in the up direction excluded outliers as the vehicle was bound on the ground.

Furthermore, to estimate the velocity at epochs where Doppler cannot be obtained, such as in tunnels, the velocity at all epochs was interpolated from the previous and next velocity information. Here, Makima interpolation [39] was adopted rather than simple linear or spline interpolation. Makima interpolation does not produce the overshoot produced by spline interpolation and it is suitable for velocity interpolation. Finally, this estimated velocity was adopted in the next position-optimization step.

## 7. Position-Estimation Step

**Figure 7:**
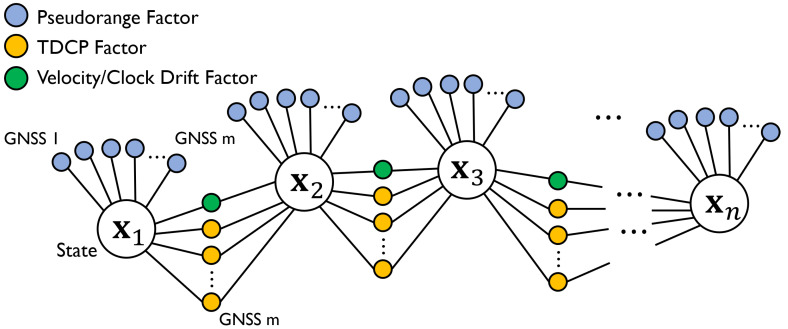
Graph structure of proposed method in position-estimation step. Pseudorange, TDCP, and velocity/clock drift factors were adopted.

In the position-estimation step, the state X estimated in epoch *i* was the 3D position relative to the initial position in ECEF coordinates and multi-GNSS clock biases. The state X can be expressed as:

(12) $$X_{i}=\left[\begin{array}{c} r_{i}\\ t_{i} \end{array}\right]$$

(13) $$r_{i}={\left[\begin{array}{ccc} x_{i} & y_{i} & z_{i} \end{array}\right]}^{T}\;t_{i}={\left[\begin{array}{cccccc} t_{\mathrm{gpsL}1,i} & t_{\mathrm{glo},i} & t_{\mathrm{galL}1,i} & t_{\mathrm{bds},i} & t_{\mathrm{gpsL}5,i} & t_{\mathrm{galL}5,i} \end{array}\right]}^{T}$$

where $t_{\mathrm{gpsL}1,i}$ denotes the receiver clock bias computed from GPS L1 signals, and $t_{\mathrm{glo},i}$, $t_{\mathrm{galL}1,i}$, $t_{\mathrm{bds},i}$, $t_{\mathrm{gpsL}5,i}$, and $t_{\mathrm{galL}5,i}$ represent the system biases including the time bias of the GLONASS, Galileo, and BeiDou relative to the GPS L1 signal, respectively. The state was defined by the difference in the initial values of the 3D position and clock bias for linearization. Figure 7 presents the proposed graph structure. Three types of factors, pseudorange, TDCP, and velocity/clock drift, were used.

### 7.1. Velocity/Clock Drift Factor

The velocity/clock drift factor is the relative constraint between time-series variable nodes. In the previous step, the 3D velocity and clock drift at all epochs were estimated based on Doppler observations; hence, the estimated velocity and clock drift were adopted as loose constraints between sequential states in the position-estimation step. If TDCP observations are not available, this velocity/clock drift factor will be the only constraint between sequential states. The error function of the velocity/clock drift factor is denoted as:

(14) $$e_{v,i}=\left(X_{i+1}-X_{i}\right)-\left(\frac{{\tilde{V}}_{i}+{\tilde{V}}_{i+1}}{2}\right)\Delta t$$

where $\tilde{V}$ denotes the velocity and clock drift estimated in the previous step, with “0” added to match the dimension of X. The velocity/clock drift factor was added between all nodes, such that, even in epochs where the pseudorange or TDCP was not observed at all (such as in a tunnel), the position was estimated based on the velocity estimated in the previous step.

### 7.2. Pseudorange Factor

In the pseudorange factor, the pseudorange compensated using the pseudorange error computed at a base-station was adopted to estimate the absolute position. The GNSS pseudorange of satellite *k* in the *i*-th epoch $\rho_{i}^{k}$ can be modeled as follows.

(15) $$\rho_{i}^{k}=r_{i}^{k}+t_{i}-\delta T_{i}^{k}+I_{i}^{k}+T_{i}^{k}$$

where $r_{i}^{k}$ denotes the geometric satellite-to-receiver distance, which is calculated using the initial node. Further, $\delta T_{i}^{k}$, $I_{i}^{k}$, and $T_{i}^{k}$ represent the clock bias of the satellite, ionospheric delay, and tropospheric delay, respectively.

To completely eliminate the satellite orbit, clock, tropospheric, and ionospheric delays in the smartphone pseudorange observations, the GNSS pseudorange error at the GNSS base-station was calculated to correct the pseudorange. The error function of the pseudorange factor is represented as follows.

(16) $$e_{\mathrm{pr},i}^{k}=H_{p,i}^{k}X_{i}-\left(\rho_{i}^{k}-r_{i}^{k}-\varepsilon_{i}^{k}\right)$$

where $\varepsilon_{i}^{k}$ denotes the pseudorange correction value, including the tropospheric and ionospheric delay, and the clock and orbit error of the satellite, calculated at a base-station whose position is known. Here, the measurement matrix $H_{p,i}^{k}$ can be formulated as:

(17) $$H_{p,i}^{k}=\left[\begin{array}{ccccccc} u_{i}^{k} & 1 & \delta_{\mathrm{glo},i}^{k} & \delta_{\mathrm{gal},i}^{k} & \delta_{\mathrm{bds},i}^{k} & \delta_{\mathrm{gpsL}5,i}^{k} & \delta_{\mathrm{galL}5,i}^{k} \end{array}\right]$$

where $\delta_{\mathrm{glo},i}^{k}$, $\delta_{\mathrm{gal},i}^{k}$, $\delta_{\mathrm{bds},i}^{k}$, $\delta_{\mathrm{gpsL}5,i}^{k}$, and $\delta_{\mathrm{galL}5,i}^{k}$ are equal to “1” when the *k*-th GNSS measurement is GLONASS, Galileo, or BeiDou, respectively. For GSDC 2022, we selected the GNSS base-station closest to the trajectory from the NOAA CORS Network and utilized it as the base GNSS station. Regarding the information matrix of the pseudorange factor, we used an elevation angle-based error model and the Doppler factor.

### 7.3. TDCP Factor

Both Doppler and TDCP can be used to estimate the velocity. However, Doppler observations from smartphones are noisier than those from commercial GNSS receivers and velocity computed from TDCP measurement is significantly more accurate than those calculated from Doppler. The TDCP measurement $\Delta \Phi_{i}^{k}$ between sequential epochs *i* and i+1 is expressed as follows:

(18) $$\lambda \Delta \Phi_{i}^{k}=\lambda \left[\Phi_{i+1}^{k}-\Phi_{i}^{k}\right]≃\Delta r_{i}^{k}+\Delta t_{i}$$

where λ, $\Phi_{i}^{k}$, and $r_{i}^{k}$ denote the signal wavelength, measured carrier phase in cycles, and satellite-receiver geometric distance, respectively. Δ represents the operator that computes the time difference. If the time difference is short, the ionospheric and tropospheric delays and satellite orbital clock errors in the carrier phase can be canceled. Here, $\lambda \Delta \Phi_{i}^{k}$ represents the exact receiver–satellite distance change between epochs *i* and i+1.

The error function of TDCP factor is as follows.

(19) $$e_{\mathrm{td},i}^{k}=H_{p,i}^{k}\left(X_{i+1}-X_{i}\right)-\left(\lambda \Delta \Phi_{i}^{k}-\Delta L_{i}^{k}\right)$$

Here, $\Delta L_{i}$ represents the change in distance owing to the satellite motion from the antenna position.

However, although TDCP is accurate, its availability is lower than the Doppler owing to cycle slip and half-cycle ambiguity challenges. If the carrier phase with cycle slip is not completely excluded, the TDCP factor will introduce relative position errors. Here, we address this cycle-slip problem with robust optimization using the M-estimator.

### 7.4. Optimization

The final objective function to be optimized, using all the factors, is presented as follows.

(20) $$\hat{X}=\underset{X}{\mathrm{argmin}}\sum_{i}{∥e_{v,i}∥}_{\Omega_{v,i}}^{2}+\sum_{i}\sum_{k}{∥e_{\mathrm{pr},i}^{k}∥}_{\Omega_{\mathrm{pr},i}^{k}}^{2}+\sum_{i}\sum_{k}{∥e_{\mathrm{td},i}^{k}∥}_{\Omega_{\mathrm{td},i}^{k}}^{2}$$

Similar to that in the velocity estimation step, robust estimation by the M-estimator with the Huber function was applied to the pseudorange and TDCP factors. Because the pseudorange observations contain multipath errors and the TDCP observations contain cycle slips, the M-estimator was adopted to exclude outliers. The hyperparameters of the Huber function were determined by trial and error. The smartphone position optimized in the position-estimation step was directly used as the final estimate.

## 8. Evaluation and Discussion

### 8.1. Evaluation Using Training Data

#### 8.1.1. Evaluation of Positioning Accuracy

The training dataset, for which reference locations were provided, was used to evaluate the positioning accuracy of the proposed method. The GSDC 2022 training dataset contains 170 smartphone trajectories on 62 different routes. From these trajectories, we constructed a dataset for evaluation considering the following points:

1 Exclude runs for which the ground truth does not include altitude. Although the final score is determined by the horizontal positioning error, runs without ground truth for altitude were excluded. This is because we cannot compute position and velocity references in the ECEF coordinates.
2 Exclude runs for which the carrier phase has not been obtained. The HardwareClockDiscontinue flag in the log is only reported on Google Pixel 4, which includes some runs for which the carrier phase has not been obtained correctly. Because TDCP cannot be used in these cases, they were excluded from the evaluation.
3 During the competition, a participant pointed out that the ground truth for some runs was not sufficiently accurate. The competition host then made an announcement and published a list of the inaccurate ground truths. The runs containing these inaccurate ground truths were eliminated.

Using the above process, the proposed method is evaluated on the 55 runs extracted from the training dataset.

**Figure 8:**
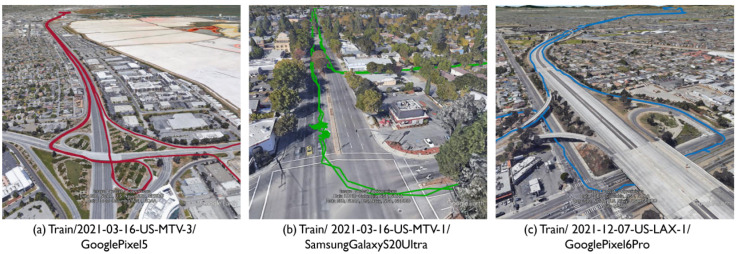
Driving trajectory of three vehicles extracted from the training dataset for evaluation (**a**) highway environment, (**b**) street driving, and (**c**) driving with GNSS blockage for a long period of time.

Three runs were selected from the entire evaluation data; the trajectories of each baseline are illustrated in Figure 8. Figure 8a–c present the data from the highway runs, street driving, and the area around Los Angeles, including the GNSS signal blockage for a long period of time, respectively.

**Figure 9:**
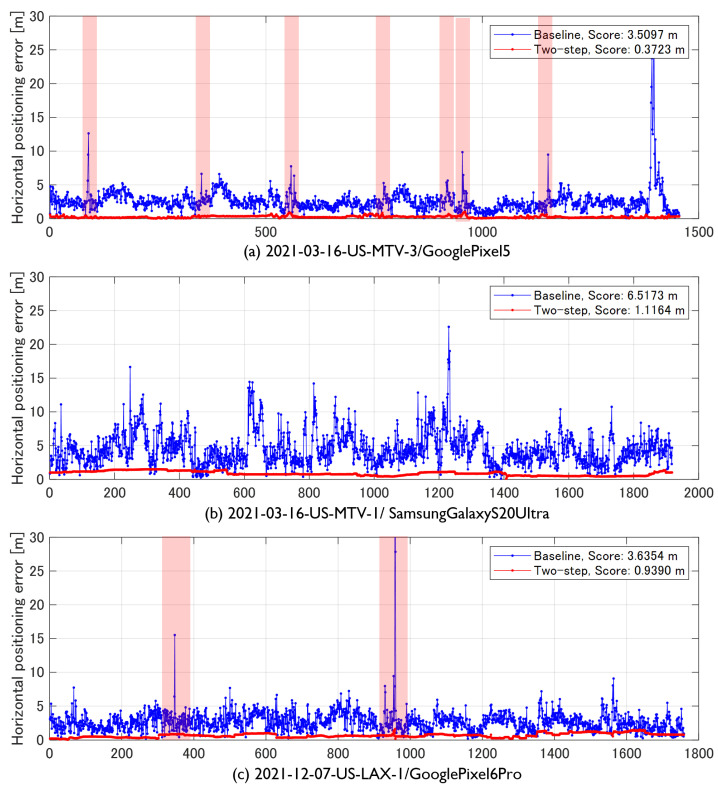
Comparison of horizontal positioning error between baseline (blue line) and proposed method (red line).

Figure 9 presents the time-series horizontal positioning error. The blue line indicates the baseline position error and the red line indicates the proposed method. The colored areas in Figure 9 are the sections that traveled under the elevated tracks. In highway driving (Figure 9a), the TDCP factor estimated the relative position with high accuracy and the pseudorange factor corrected the absolute position. Furthermore, the baseline position sometimes exhibited an abrupt error of approximately 10 m, which occurred when the vehicle went under an elevated road while driving on the highway. However, the proposed method suppressed the increase in error by interpolating the velocity estimated from Doppler. Figure 9b presents the positioning error of street driving. The pseudorange and carrier-phase noise were large and the position-estimation accuracy was worse than that of highway driving. Furthermore, the baseline exhibited frequent position errors exceeding 10 m; however, the proposed method could estimate the accurate position. Figure 9c presents the positioning error of driving in the Los Angeles area. Although the error increased at the point where the vehicle went under the overpass, the proposed method significantly suppressed the increase in error when compared to the baseline.

**Figure 10:**
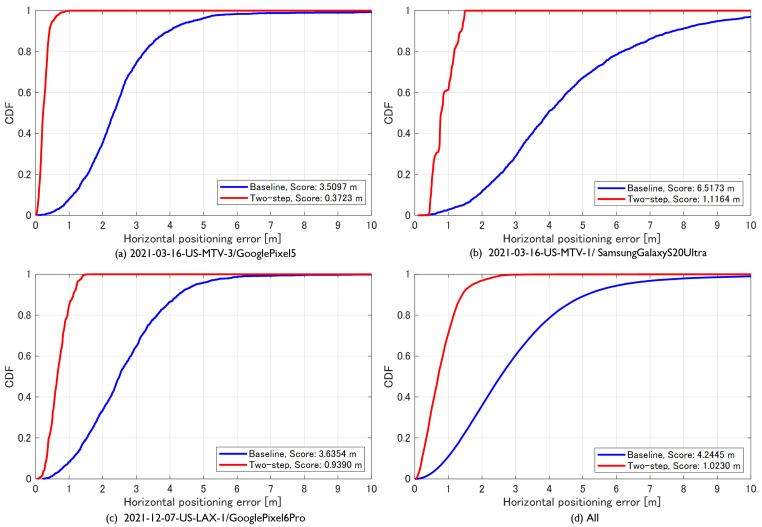
Comparison of horizontal cumulative distribution function (CDF) for each run and the entire evaluation dataset. The blue and red lines represent the baseline and proposed method, respectively.

Figure 10 illustrates the horizontal cumulative distribution function (CDF) for each run and the entire evaluation dataset. Table 2 presents the 50 percentile error, the 95 percentile error, and the score calculated using Equation (3). The score was 0.372 m in the highway driving case, thus implying the achievement of decimeter accuracy. The score was 1.116 m in the street driving data. The final score for all runs included in the evaluation dataset was 1.023 m. The proposed method significantly improved the baseline scores provided by the host, thereby achieving decimeter accuracy when evaluated only on the highway data, but only slightly more than 1 m when evaluated on all runs.

#### 8.1.2. Evaluation of Two-Step Optimization

The key contribution of the proposed method is that the velocity and position-estimation steps are separated and the position is estimated in a two-step optimization. To evaluate the effectiveness of the two-step optimization, we compared it with a one-step optimization method that simultaneously estimates position and velocity. The one-step optimization method is based on an implementation based on GSDC 2021, which adds 3D position, velocity, and receiver clock bias drift to the estimated state [36]. The GNSS observations used are completely equivalent to those of the two-step optimization method and equivalent parameters, such as the information matrix of the observations, were adopted.

**Figure 11:**
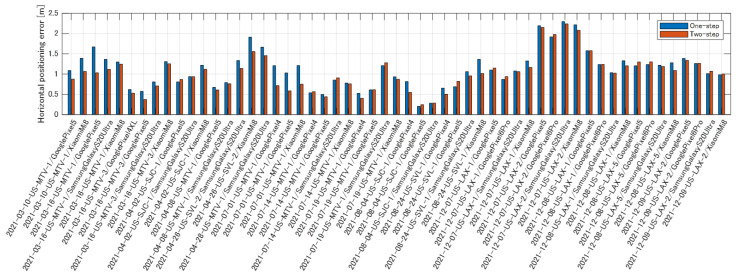
Comparison of positioning error between one-step and two-step optimization.

Figure 11 compares the scores of the one-step and two-step optimizations for each run in the evaluation data. Figure 11 demonstrates that the proposed two-step optimization method outperforms the former in almost all runs. Table 3 presents the scores of the one-step and two-step optimization methods for all runs. The score of the one-step optimization is 1.122 m, thus indicating that the proposed two-step optimization method exhibits better performance.

### 8.2. Evaluation Using Test Data

Using the above method, the position of the smartphone was estimated and the competition was addressed. Finally, the public score for the proposed method was 1.382 m, which ranked first. The final private score was 1.229 m, which was also in the first place. Hence, the proposed method can be used to estimate the position of smartphones with high accuracy. After the competition, the scores were calculated again with the final adjusted parameters (equivalent to those used in the evaluation of the training dataset above), resulting in public and private scores of 1.364 m and 1.177 m, respectively.

### 8.3. Discussion

The proposed two-step optimization method can estimate the position of the smartphones with an accuracy of 1.229 m in the GSDC 2022 test dataset. Here, the second place score was 1.499 m. Therefore, the proposed method is substantially the most accurate method for smartphone position estimation. The RTK-GNSS, which estimates the ambiguity of the carrier phase using the double-differenced GNSS observation from the base-station, is difficult to adopt with the noisy carrier phase of a smartphone; however, the TDCP observation, which is the time difference of its own carrier phase, can estimate the relative position with high accuracy.

The proposed two-step optimization was more accurate than the one-step optimization because the velocity was estimated first using Doppler observations, which are more robust, and the outliers of the estimated velocity were rejected beforehand and interpolated for missing values, which is considered to have an effect on accuracy.

The training data with position references for machine learning were provided; however, the proposed method did not use machine learning. Therefore, the incorporation of machine learning into the graph optimization framework represents a future challenge. In addition, IMU data from smartphones were also provided; however, the method described in this paper adopts only GNSS. Although there is a time synchronization problem between the smartphone IMU and GNSS [40], the combined IMU data are expected to improve accuracy in environments where GNSS signals are more shielded.

## 9. Conclusions

This paper described a method for estimating the position of a smartphone used in GSDC 2022. The proposed method adopted factor graph optimization to estimate the entire trajectory of a smartphone by creating various factors from the smartphone’s GNSS observations. A two-step optimization method that estimates velocity and position in separate steps was proposed. The proposed method first estimated the velocity from the GNSS Doppler observation via graph optimization. The outliers of the estimated velocity were then excluded, interpolated, and subsequently used as loose constraints between states in the position-optimization step to make the position estimation more robust and accurate. Absolute constraints were added to the graph using corrected GNSS pseudoranges. In addition, the graph optimization process enabled highly accurate estimation of the 3D position of a smartphone.

Using the GSDC2022 training dataset, we compared the proposed two-step optimization with a one-step optimization that simultaneously estimates position and velocity and found that the two-step optimization improved the score from 1.12 m to 1.02 m. The results confirmed that estimating the velocity first improves the position estimation accuracy. Accordingly, from the proposed method presented at GSDC2022, the final private score was 1.229 m, which won the first place.

## Figures and Tables

**Table 1:** Score of baseline position for each course type in training dataset of GSDC 2022.

Course Type	Highway	Street	All
Score (50%) m	2.296	2.772	2.599
Score (95%) m	4.943	6.431	5.890
Mean m	3.620	4.602	4.245

**Table 2:** Comparison of positioning error between baseline and proposed method.

Course Phone	2021-03-16-US-MTV-3 GooglePixel5		2021-03-16-US-MTV-1 SamsungGalaxyS20Ultra		2021-12-07-US-LAX-1 GooglePixel6Pro		All	
	**Baseline**	**Two-Step**	**Baseline**	**Two-Step**	**Baseline**	**Two-Step**	**Baseline**	**Two-Step**
Score (50%) m	2.322	0.225	3.963	0.782	2.494	0.648	2.599	0.749
Score (95%) m	4.697	0.520	9.072	1.450	4.777	1.230	5.890	1.297
Mean m	3.510	0.372	6.517	1.116	3.635	0.939	4.245	1.023

**Table 3:** Summary of positioning error comparison between one-step and two-step optimization.

Course	All	
	**One-Step**	**Two-Step**
Score (50%) m	0.801	0.749
Score (95%) m	1.442	1.297
Mean m	1.122	1.023
